# Flow-driven left ventricular remodelling in long-term haemodialysis: a multicenter model of arteriovenous access-induced cardiac load

**DOI:** 10.1080/0886022X.2026.2622136

**Published:** 2026-02-05

**Authors:** Naoko Kyoda, Rika Ago, Shingo Fukuma, Takao Masaki

**Affiliations:** ^a^ Department of Nephrology, Shiritsu Miyoshi Chuo Byoin, Hiroshima, Japan; ^b^ Department of Nephrology, Hiroshima University Hospital, Hiroshima, Japan; ^c^ Department of Epidemiology, Disease Control and Prevention, Hiroshima University Hospital, Hiroshima, Japan

**Keywords:** Arteriovenous fistula, left ventricular hypertrophy, cardio-renal syndrome, haemodynamic load, dialysis vascular access

## Abstract

**Background:**

Arteriovenous (AV) access, an essential component of dialysis treatment, has been implicated in the development of cardiovascular abnormalities. However, whether AV access blood flow and the duration of exposure to this flow, as represented by the dialysis vintage, have independent or interactive effects on cardiac outcomes remains unclear.

**Methods:**

In this multicenter cross-sectional study, we enrolled hemodialysis patients who had undergone both transthoracic echocardiography and measurement of AV access flow, between April 2014 and January 2024. Left ventricular mass index (LVMI) was used as a surrogate marker of cardiovascular morbidity. AV access flow was quantified as brachial artery flow volume (FV) using pulsed Doppler ultrasonography. Multivariate regression models were applied to investigate the associations of FV and dialysis vintage with LVMI, and to assess potential interactions between these variables.

**Results:**

A total of 241 patients were included. The mean FV was 0.71 ± 0.29 L/min, and the median dialysis vintage was 5.0 years (interquartile range, 3.0–11.0 years). Higher FV was significantly associated with increased LVMI (mean difference = 22.83; 95% confidence interval [CI]: 0.37 to 45.29; *p* = 0.046), whereas dialysis vintage was not (mean difference = 0.51; 95% CI: −0.41 to 1.45; *p* = 0.27). Moreover, no statistically significant interaction was observed (mean difference = 0.13; 95% CI: −3.14 to 3.40; *p* = 0.94).

**Conclusion:**

AV access flow may serve as a candidate variable in future left ventricular hypertrophy prediction algorithms and cardio-renal machine learning models because of its linear and independently significant association with LVMI.

## Graphical Abstract

**Figure:**
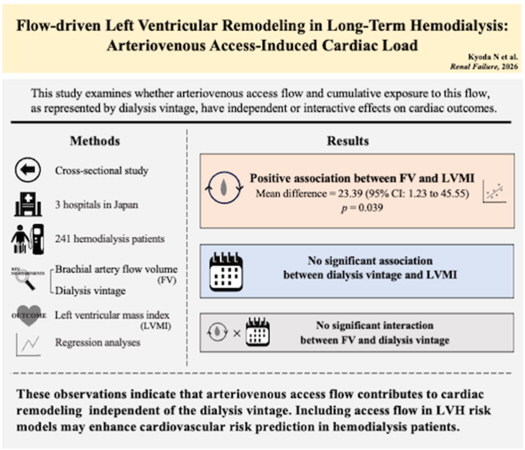

**Box.** What This Study Suggests for Future Clinical Practice and Guideline Development

What this study adds:

- Arteriovenous access flow independently contributes to left ventricular (LV) remodeling, supporting its role as a haemodynamic driver of cardiac hypertrophy in long-term hemodialysis patients.
- Dialysis duration alone does not exacerbate flow–left ventricular hypertrophy (LVH) coupling, suggesting that LV remodeling risk is more flow-dependent than exposure-time dependent once long-term hemodialysis is established.
- Existing ‘high-flow’ thresholds may require reassessment, and individualized flow targets may be needed for cardio-protection, particularly in patients demonstrating progressive LVH, despite clinically acceptable dialysis adequacy.
- These findings support the potential integration of access flow parameters into cardiovascular risk stratification models, access surveillance policies, and future interventional flow modulation trials.

## Introduction

As of 2023, approximately 350,000 individuals in Japan are receiving maintenance dialysis. Among this population, cardiovascular disease—including heart failure, cerebrovascular disease, and myocardial infarction—remains the leading cause of mortality [1]. To improve cardiovascular outcomes in dialysis patients, various therapeutic strategies have been implemented, including blood pressure control, fluid management, correction of anemia, and management of chronic kidney disease-mineral and bone disorder (CKD-MBD) [2–5]. These interventions have contributed to a steady decline in cardiovascular mortality, from 54.8% in 1988 to 29.4% in 2023 [1]. However, cardiovascular disease continues to be a major determinant of prognosis in this population, indicating that further improvements may require reevaluation of current dialysis practices.

Arteriovenous (AV) access, while essential for hemodialysis delivery, represents a non-physiological circulatory shunt and has been identified as a potential contributor to cardiovascular dysfunction [6–8]. The haemodynamic alterations induced by AV access can affect cardiac structure (e.g., left ventricular mass, LVM) and function [7–10]. Elevated AV access flow has been associated with a spectrum of complications, including high-output heart failure, pulmonary hypertension, central venous stenosis, venous hypertension, aneurysmal degeneration of arteriovenous fistulas (AVF), and access-related distal ischemia [11]. Although access ligation or flow-reduction procedures are typically considered in symptomatic patients, even low-flow AVFs have been reported to impair myocardial oxygen supply [12,13] and sustained volume overload may contribute to irreversible impairment in myocardial contractility [14]. While most hemodialysis patients use an AVF or arteriovenous graft (AVG) for vascular access, it remains unclear whether the presence of AV access flow—regardless of clinical symptoms or flow adequacy—independently influences cardiac prognosis.

The hemodialysis population is clinically heterogeneous, with variations in age, sex, comorbid conditions, dialysis vintage, blood pressure, CKD-MBD, and cardiovascular history, all of which may affect cardiac outcomes [15,16]. Notably, current Kidney Disease Outcomes Quality Initiative guidelines do not recommend routine AVF/AVG surveillance based on AV access flow measurements, citing insufficient supporting evidence [11]. Therefore, the clinical significance of AV access flow in relation to cardiac prognosis remains uncertain. Furthermore, kidney transplantation rates are markedly lower in Japan than in other developed nations. According to the Organ Procurement & Transplantation Network, 28,142 kidney transplants were performed in the United States in 2023 [17], compared with only 2,001 in Japan [18]. Even after adjusting for population size, this discrepancy suggests that Japan has a disproportionately large population of long-term dialysis patients. These individuals are chronically exposed to AV access flow, raising the question of whether dialysis duration contributes independently to adverse cardiac remodeling, or whether it amplifies the effects of AV access flow in a synergistic manner.

Left ventricular hypertrophy (LVH), most commonly assessed *via* echocardiographic measurement of LVM, is an established predictor of cardiovascular morbidity and mortality in patients with end-stage renal disease [19–21]. In this study, we investigated the association between AV access flow and cardiac remodeling, using LVM as a surrogate marker of cardiovascular risk. Additionally, we explored whether dialysis vintage modifies the relationship between AV access flow and LVM.

## Materials and methods

### Study design and setting

This multicenter cross-sectional study was conducted at three institutions—Miyoshi Central Hospital, Miyoshi Medical Association Hospital, and Fuchu Kita City Hospital—all located in the northern region of Hiroshima, Japan. Each facility provides maintenance hemodialysis services. Patients undergoing maintenance hemodialysis between April 2014 and January 2024 were considered for inclusion.

### Ethics

This study was conducted in accordance with the principles of the Declaration of Helsinki. The study protocol was reviewed and approved by the Ethics Committee of Miyoshi Central Hospital (approval number: 060306-1). Written or verbal informed consent was not obtained because of the retrospective nature of the study. Instead, an opt-out approach was used in accordance with institutional guidelines: study details were posted on hospital bulletin boards, and patients were given the opportunity to review the information and decline participation if desired.

### Eligibility criteria

**Figure 1.**
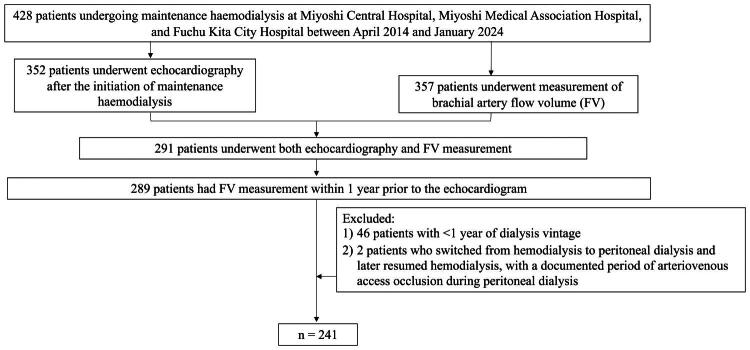
Study flowchart of maintenance hemodialysis patients evaluated for arteriovenous access flow and left ventricular mass index (LVMI).

Patients were eligible for inclusion if they met the following criteria: (ⅰ) age greater than 18 years; (ⅱ) presence of an AVF or AVG suitable for hemodialysis; and (ⅲ) availability of both echocardiographic data and AV access flow measurements obtained within 1 year prior to the echocardiogram. For patients with multiple eligible datasets, the most recent was selected. To avoid bias from immature AVF development and preexisting cardiac abnormalities, patients with a dialysis vintage of less than 1 year were excluded. This approach was designed to minimize inclusion of patients with concurrently low access flow and baseline LVH (Figure 1).

### Data collection methods

Clinical and demographic data were extracted from electronic medical records, including age, sex, height, weight, comorbidities, medication use, dialysis-related variables, blood pressure, laboratory test results (hemoglobin, creatinine, phosphorus, calcium, albumin, intact parathyroid hormone, β_2_-microglobulin, total cholesterol, and triglycerides), and echocardiographic measurements. Blood pressure data were derived from the average of pre-dialysis readings obtained at the start of the week across four dialysis sessions preceding LVMI assessment.

### Vascular access flow assessment

AV access blood flow was assessed *via* brachial artery flow volume (FV, L/min), measured using pulsed Doppler ultrasonography. All measurements were conducted with ultrasound systems from Toshiba Medical Systems Corporation (now Canon Medical Systems Corporation, Otawara, Tochigi, Japan), including the Xario 100 (TUS-X100), Viamo (SSA-640A) with a 12 MHz linear probe (PLU-1204BT), and the Aplio MX (SSA-780A) with a 7.5 MHz linear probe (PLT-704AT). Examinations were performed with patients in the supine position during dialysis using the pulsed-wave Doppler method.

Throughout the study period, all measurements were obtained by trained clinical engineers (medical engineers) in dialysis units using a standardized protocol (angle correction ≤60° and sampling at a straight segment of the brachial artery). Flow volume was measured with the blood pump and dialysate pump temporarily stopped, and then automatically calculated by the ultrasound software using the formula: FV = time-averaged flow velocity (cm/s) × cross-sectional area (cm^2^) × 60 ÷ 1,000 [22], and the results are expressed in L/min. A formal inter-device validation of FV measurements was not performed. To minimize measurement variability caused by access-related factors such as stenosis or repeated angioplasty, all available prior FV measurements were averaged. Cases with suspected or confirmed access occlusion, defined as FV < 0.1 L/min or resistance index > 1, were excluded from the analysis.

### Echocardiography

All patients underwent transthoracic echocardiography performed by cardiologists, either on dialysis or non-dialysis days. Indications for echocardiographic evaluation included routine annual screening, preoperative assessment, and clinical suspicion of cardiac disease. Standard parasternal long-axis views were used to measure interventricular septal thickness (IVST), posterior wall thickness (PWT), and left ventricular end-diastolic diameter (LVEDD). LVM and body surface area (BSA) were subsequently calculated as follows using the validated formulas:

$$\text{LVM}=0.8\times \left[1.04\times {\left(\text{IVST}+\text{LVEDD}+\text{PWT}\right)}^{3}-{\left(\text{LVEDD}\right)}^{3}\right]+0.6\left[23\right]$$

$$\text{BSA}=\text{height}{\left(\text{cm}\right)}^{0.725}\times \text{body weight}{\left(\text{kg}\right)}^{0.425}\times 0.007184\left(\text{Du Bois formula}\right)\left[24\right]$$

LVMI was derived by dividing LVM by BSA [23]. LVH was defined as an LVMI > 115 g/m^2^ in men and > 95 g/m^2^ in women [23]. In patients who underwent echocardiography on a non-dialysis day, the pre-dialysis weight from the subsequent session was used to calculate BSA. In examinations performed immediately before or after dialysis, the corresponding pre- or post-dialysis body weight was used [24].

### Statistical analysis

No formal sample size estimation or correction for multiple comparisons was conducted because of the exploratory nature of the study. Categorical variables are presented as counts and percentages, and continuous variables are reported as mean (standard deviation) or median (interquartile range [IQR]), as appropriate. Linear regression models were used to evaluate the associations between FV, dialysis vintage, and LVMI. Univariate analyses were first conducted to assess individual variable associations, followed by multivariate linear regression (Model 1), with LVMI as the dependent variable and FV and dialysis vintage as the primary independent variables. Covariates included age, sex, diabetes mellitus, antihypertensive medication, systolic blood pressure, phosphate, calcium, albumin, dyslipidaemia, the modality of dialysis (hemodialysis or online haemodiafiltration), and proximity to dry weight [15,16,25,26] to adjust for potential confounding. An interaction term between FV and dialysis vintage (FV × dialysis vintage) was added to the multivariate model (Model 2) to assess potential effect modification. Both FV and dialysis vintage were mean-centered to enhance interpretability. Residuals were visually assessed for normality using histograms. All variables had complete data; thus, no imputation was necessary. Because the objective was to examine associations rather than prediction, model fit was not evaluated. Statistical analyses were performed using R software (version 4.4.2; R Foundation for Statistical Computing, Vienna, Austria). Data handling and visualization were conducted using the following packages: *readxl*, *rms*, *dplyr*, *car*, and *ggplot2*.

## Results

### Study population

Figure 1 shows a flow diagram detailing participants’ enrollment, exclusions, and final inclusion. Of the patients screened, 241 met all eligibility criteria and were included in the final analysis cohort.

Baseline demographic and clinical characteristics are summarized in Table 1. The median age of the study population was 77 years (IQR, 69.5–84.5 years), and 58% were men. Comorbidities included diabetes mellitus, dyslipidaemia, and cardiovascular disease in 49%, 52%, and 25% of patients, respectively. The median dialysis vintage was 5.0 years (IQR, 3.0–11.0 years). The mean pre-dialysis systolic blood pressure, measured at the beginning of the week, was 143.1 ± 24.9 mmHg, and the mean diastolic blood pressure was 72.9 ± 12.8 mmHg. A laboratory examination showed a mean hemoglobin level of 11.0 ± 1.4 g/dL, a mean phosphate concentration of 4.5 ± 1.4 mg/dL, a mean calcium level of 8.6 ± 0.7 mg/dL, and a mean serum albumin concentration of 3.1 g/dL. The mean AV access FV was 0.71 ± 0.29 L/min, with a median of 17.0 FV measurements per patient (IQR, 9.0–33.0). The mean LVMI was 141.4 ± 48.6 g/m^2^. A total of 78% of the patients had LVH. Table 2 shows the clinical parameters of patients on hemodialysis with and without LVH.

**Table 1.** Baseline clinical, dialysis, and cardiovascular parameters in long-term hemodialysis patients.

Characteristic	Value	
Number of patients	241	
Age (years)	77	(69.5–84.5)
Male sex, *n* (%)	140	(58)
BMI (kg/m^2^)	21.8	(4.1)
Comorbidities, *n* (%)		
Hypertension	221	(92)
Diabetes mellitus	118	(49)
Dyslipidaemia	125	(52)
Cardiovascular disease	61	(25)
Ever smoked or current smoker	91	(38)
Antihypertensive medication, *n* (%)		
No medication	177	(73)
Calcium channel blocker	110	(46)
ACE inhibitor or angiotensin II receptor blocker	86	(36)
Angiotensin receptor-neprilysin inhibitor	4	(2)
α blocker	35	(15)
β blocker	68	(28)
Methyldopa	8	(3)
Diuretic	63	(26)
Dialysis-related parameters		
Hemodialysis vintage (years)	5.0	(3.0–11.0)
Previous peritoneal dialysis, *n* (%)	42.0	(2)
Modality of dialysis		
Hemodialysis	164	(68)
Online haemodiafiltration	76	(32)
Blood flow (mL/min)	230	(200–250)
FV (L/min)	0.71	(0.29)
Number of access flow measurements (times)	17.0	(9.0–33.0)
Blood pressure (mmHg)		
Systolic	143.1	(24.9)
Diastolic	72.9	(12.8)
Pre-dialysis laboratory parameters		
Hemoglobin (g/dL)	11.0	(1.4)
Creatinine (mg/dL)	8.0	(2.4)
Phosphate (mg/dL)	4.5	(1.4)
Calcium (mg/dL)	8.6	(0.7)
Albumin (g/dL)	3.1	(0.6)
Intact PTH (pg/mL)	146.0	(98.0)
β_2_-microglobulin(mg/L)	29.0	(6.7)
Total cholesterol (mg/dL)	142.2	(33.0)
Triglyceride (mg/dL)	88.8	(50.1)
Echocardiographic measurements		
LVEF (%)	64.1	(12.6)
IVST (mm)	11.2	(2.5)
PWT (mm)	10.8	(2.5)
LVDd (mm)	45.7	(7.9)
LV mass (g)	213.5	(76.1)
LV mass index (g/BSA)	141.4	(48.6)
LV hypertrophy, *n* (%)	187	(78)
Significant AS^a^	28	(12)
Significant AR^a^	16	(7)
Significant MR^a^	35	(15)
Post AVR or TAVI	7	(3)

Data are presented as mean (standard deviation), median (interquartile range), or number (percentage), as appropriate.

^a^ Defined as moderate and severe valvular disease.

BMI: body mass index; FV: brachial artery flow volume; LVEF: left ventricular ejection fraction; IVST: interventricular septum thickness; PWT: posterior wall thickness; LVDd: left ventricular end-diastolic diameter; LV: left ventricular; BSA: body surface area; AS: aortic stenosis; AR: aortic regurgitation; MR: mitral regurgitation; AVR: aortic valve replacement; TAVI: transcatheter aortic valve implantation

**Table 2.** Baseline clinical, dialysis, and cardiovascular parameters according to the presence of LVH in long-term hemodialysis patients.

Parameter	LVH (*n* = 187)		without LVH (*n* = 54)	
Age (years)	77	(72.0–86.0)	75	(65.6–81.3)
Male sex, *n* (%)	98	(52)	42	(78)
Hypertension, *n* (%)	174	(93)	47	(87)
Diabetes mellitus, *n* (%)	89	(48)	29	(54)
Dyslipidaemia, *n* (%)	99	(53)	26	(48)
Systolic blood pressure (mmHg)	144.3	(23.5)	138.4	(28.7)
Antihypertensive medication, *n* (%)	48	(26)	16	(30)
Hemodialysis vintage (years)	5	(3–11)	5	(3–9)
Modality of dialysis, *n* (%)				
Hemodialysis	133	(71)	27	(50)
Online haemodiafiltration	54	(29)	27	(50)
FV (L/min)	0.71	(0.30)	0.68	(0.26)
Hemoglobin (g/dL)	11.0	(1.4)	10.8	(1.6)
Phosphate (mg/dL)	4.5	(1.3)	4.4	(1.4)
Calcium (mg/dL)	8.6	(0.8)	8.5	(0.7)
Albumin (g/dL)	3.2	(0.6)	3.1	(0.7)
Significant valvular disease (AS/AR/MR),^a^ *n* (%)	60	(32)	6	(11)

Data are presented as mean (standard deviation), median (interquartile range), or number (percentage), as appropriate.

^a^ Defined as moderate and severe valvular disease.

LVH: left ventricular hypertrophy; FV: brachial artery flow volume; AS: aortic stenosis; AR: aortic regurgitation; MR: mitral regurgitation; AVR: aortic valve replacement

### Association of FV and dialysis vintage with LVMI and their interaction

**Figure 2.**
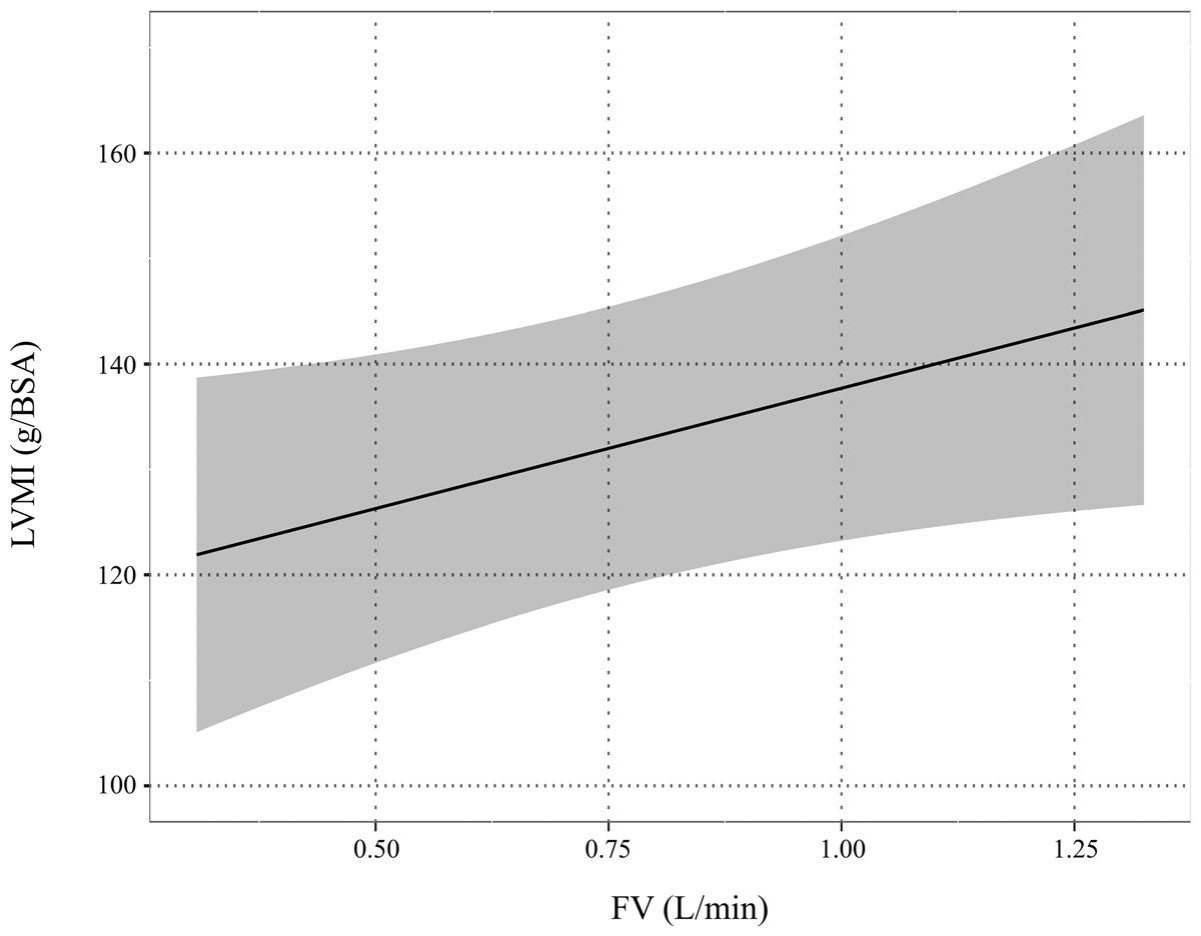
Relationship between arteriovenous access flow volume (FV) and left ventricular mass index (LVMI) demonstrating a flow-dependent remodeling pattern in hemodialysis patients (Model 1).

The univariate and multivariate analyses are shown in Table 3. In the multivariate regression model (Model 1), higher FV was independently associated with increased LVMI (mean difference = 22.83; 95% confidence interval [CI]: 0.37 to 45.29; *p* = 0.046) (Figure 2). By contrast, dialysis vintage was not significantly associated with LVMI (mean difference = 0.51; 95% CI: −0.41 to 1.45; *p* = 0.27). In a further multivariate model incorporating an interaction term between FV and dialysis vintage (Model 2), no statistically significant interaction was observed (mean difference = 0.13; 95% CI: −3.14 to 3.40; *p* = 0.94).

**Table 3.** Multivariate associations of arteriovenous access flow and dialysis vintage with LVMI.

	Univariate model			Multivariate Model 1			Multivariate Model 2		
Independent variables	β (95％ CI)		*p*-Value	β (95% CI)		*p*-Value	β (95% CI)		*p*-Value
FV	8.89	(−12.33, 30.12)	0.41	22.83	(0.37, 45.29)	0.046	22.69	(−0.07, 45.45)	0.05
Hemodialysis vintage	0.08	(−0.83, 0.99)	0.86	0.51	(−0.41, 1.45)	0.27	0.52	(−0.41, 1.45)	0.27
Covariates									
Age				1.31	(0.76, 1.87)	<0.001	1.31	(0.75, 1.88)	<0.001
Male sex				−0.74	(−7.01, 5.52)	0.81	−0.75	(−7.03, 5.53)	0.81
Diabetes mellitus				−1.95	(−8.36, 4.46)	0.27	−1.96	(−8.38, 4.47)	0.55
Antihypertensive medication				0.98	(−6.27, 8.24)	0.79	1.00	(−6.28, 8.27)	0.79
Systolic blood pressure				0.14	(−0.13, 0.40)	0.31	0.13	(−0.13, 0.40)	0.31
Hemoglobin				−2.01	(−6.73, 2.70)	0.40	−2.02	(−6.74, 2.71)	0.40
Phosphate				0.56	(−4.35, 5.47)	0.82	0.55	(−4.40, 5.47)	0.83
Calcium				0.38	(−8.22, 8.99)	0.93	0.37	(−8.27, 9.00)	0.93
Albumin				−0.57	(−14.27, 13.14)	0.93	−0.55	(−14.30, 13.20)	0.94
Dyslipidaemia				−7.04	(−13.59, −0.50)	0.035	−7.01	(−13.61, −0.41)	0.037
Online haemodiafiltration				8.17	(−5.19, 21.55)	0.23	8.18	(−5.22, 21.58)	0.23
Proximity to dry weight				2.73	(−1.75, 7,21)	0.23	2.72	(−1.77, 7.22)	0.23
Interaction term									
FV × hemodialysis vintage							0.13	(−3.14, 3.40)	0.94

Model 2 used mean-centered values of FV and dialysis vintage and included their interaction term.

LVMI: left ventricular mass index; FV: brachial artery flow volume

## Discussion

This study demonstrated a significant positive association between AV access FV and LVMI in patients undergoing maintenance hemodialysis, whereas dialysis vintage was not independently associated with LVMI. Furthermore, no significant interaction between FV and dialysis vintage was observed. To our knowledge, this is the first study to evaluate the independent and interactive effects of AV access flow and dialysis vintage on LVMI in a long-term maintenance hemodialysis population.

Previous investigations have explored the effects of AVF creation on the development of LVH. Prospective observational studies have reported increases in LVMI of approximately 5.1 g/m^2^ and a 13% rise in LVM within 6 weeks following AVF creation in patients with advanced chronic kidney disease [27,28]. In addition, the mean increase in LVMI was greater in the high-flow group than in the low-flow group. The present findings are consistent with these earlier reports, supporting the notion that AV access blood flow may contribute to the development of LVH. However, prior studies primarily examined the progression of LVH in patients during the perioperative period before and after AVF creation. Conversely, the current study focused on patients who had been receiving maintenance hemodialysis for more than 1 year, thereby offering insights into longer-term cardiac remodeling. Additionally, while many earlier studies used prospective designs, they often lacked multivariable adjustments for confounding variables. Although the present analysis was retrospective, the use of multivariate regression models controlling for key clinical covariates enhances the robustness and interpretability of our findings.

Initially, we hypothesized that the relationship between FV and LVMI could be either linear or nonlinear. Previous studies in hemodialysis patients have demonstrated a positive linear association between increased preload and LVMI [27,29–31]. In contrast, a study investigating the association between fluid overload and LVH in patients with chronic kidney disease not receiving dialysis reported that the odds ratio for LVH increased with excess fluid volume but plateaued beyond a certain threshold [32]. These findings suggested that LVH resulting from increased venous return might follow either a linear or nonlinear trajectory. In the present study, visual inspection of the scatterplot depicting FV against LVMI supported a linear association; accordingly, we employed a linear model for the primary analyses. Notably, another study in dialysis patients reported that the relationship between access blood flow and cardiac output was best characterized by a third-order polynomial regression model, with a cutoff value of 2.2 L/min for access blood flow. In that model, the slope remained flat below 2.2 L/min and increased sharply beyond this threshold [8]. In our cohort, no patients exhibited access flow exceeding 2.2 L/min, and cases with markedly elevated cardiac output were not included. Therefore, a nonlinear association, such as a plateau in LVMI, may not have been detectable within the observed flow volume range. Nonetheless, the finding that FV and LVMI maintained a positive linear relationship even within this restricted range suggests that myocardial remodeling may progress despite stable cardiac output. Future investigations that include patients with higher access flow rates may help elucidate potential nonlinear patterns, and this remains an important avenue for further research.

The potential association between dialysis duration and LVH has also been examined in previous studies. One cross-sectional observational study reported a positive association between longer dialysis vintage and the likelihood of developing LVH [33]. Another prospective study of 596 incident hemodialysis patients without prior cardiac disease found that 62% experienced an increase in LVMI after 18 months, with longer dialysis duration independently associated with LVMI elevation in multivariable analysis [34]. However, these studies largely focused on patients during the initial phases of dialysis treatment. By contrast, our cohort included individuals with longer dialysis vintage than previously examined populations. While the high prevalence of LVH among dialysis patients is well established [35–38], the extent to which prolonged dialysis contributes to progressive worsening of LVH in patients with preexisting hypertrophy remains uncertain. Our cross-sectional analysis did not identify a significant association between dialysis vintage and LVMI, which suggested a plateau effect in the progression of LVH over time. Nevertheless, given the inherent limitations of cross-sectional designs, causality cannot be inferred. Prospective longitudinal studies are warranted to clarify the temporal relationship between dialysis vintage and cardiac remodeling.

Although AV access flow and dialysis duration have both been suggested as independent contributors to LVH [27,28,33,34], to our knowledge, no previous studies have examined their potential interactive effects. Therefore, we hypothesized that the linear association between FV and LVMI is relatively modest in patients with a shorter dialysis vintage, whereas in those with a longer vintage, the increase in LVMI per unit increase in FV is steeper. Consequently, the dialysis vintage modifies (interacts with) the relationship between FV and LVMI. In our analysis, however, no significant interaction between FV and dialysis vintage was detected. There are several possible explanations for this finding. First, the absence of statistical significance does not necessarily indicate the absence of a true biological interaction. The statistical power required to detect interaction terms is generally lower than that for main effects. Additionally, our sample size, together with the observed distributions of FV and dialysis vintage, may have been insufficient to detect an interaction of clinically relevant magnitude. Second, the ranges of FV and dialysis vintage in this cohort were relatively limited, and extreme combinations of very high FV and a very long dialysis vintage were uncommon, which may have further reduced our ability to identify an interaction. Third, methodological factors may have attenuated any interaction effect. We restricted the analysis to patients who had at least one FV measurement within 1 year before assessing LVMI and retrieved all available historical FV values from the medical records to calculate a mean FV. Therefore, we took into account long-term variation in access flow, including changes related to repeated percutaneous transluminal angioplasty and access reconstruction. Nevertheless, FV was not measured at regular intervals throughout the entire dialysis vintage, and the number and timing of measurements varied between patients. Consequently, this mean FV may not fully capture cumulative exposure over the whole dialysis history. In addition, patients with suspected access problems are more likely to undergo repeated FV assessments, and such differences in measurement schedules could introduce information bias. Furthermore, inherent variability in ultrasound-based FV measurements, including possible inter-device variability, may have biased the estimated interaction effect toward the null. Finally, in this population, the effect of AV access flow on LV remodeling may have been largely independent of the dialysis duration. An example of this possibility is if most flow-related structural changes occur relatively early after creating access and subsequently plateau. Future studies with larger sample sizes, more detailed data on the access history and longitudinal LV remodeling, and analytic approaches capable of modeling non-linear and time-dependent effects are required to clarify whether, and to what extent, the dialysis duration modifies the association between FV and LVMI.

**Figure 3.**
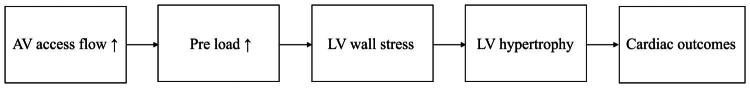
Higher arteriovenous (AV) access flow increases venous return and left ventricular (LV) preload, leading to increased LV wall stress and subsequent LV hypertrophy/remodeling. This situation may ultimately contribute to adverse cardiovascular outcomes.

**Figure 4.**
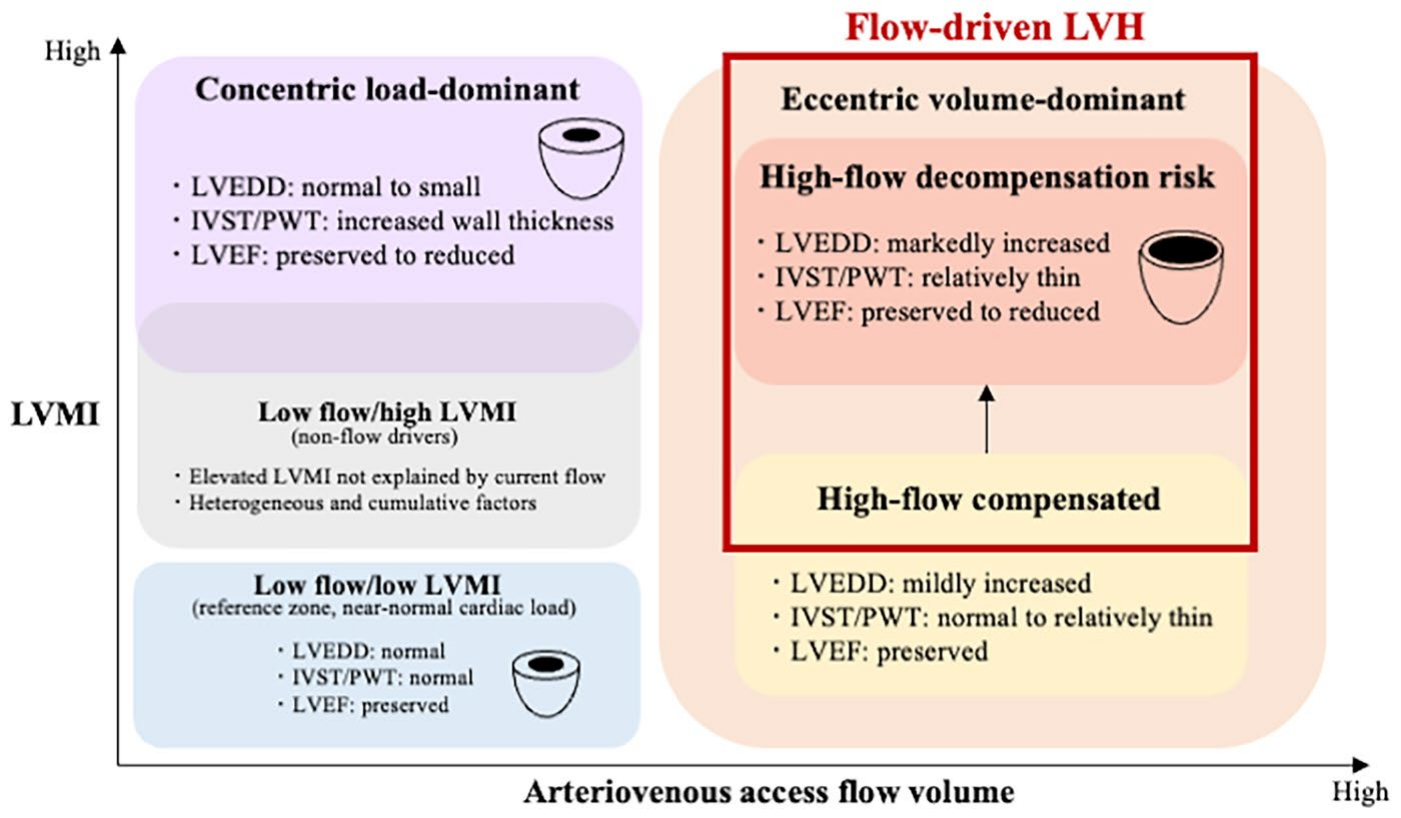
Flow–cardiac load phenotype map integrating arteriovenous access flow volume, the LVMI, and echocardiographic features. The framework primarily distinguishes between ‘eccentric volume-dominant’ and ‘concentric load-dominant’ remodeling patterns and provides a practical taxonomy for flow-driven left ventricular remodeling. LVMI, left ventricular mass index; LVEDD, left ventricular end-diastolic diameter; IVST, interventricular septal thickness; PWT, posterior wall thickness; LVEF, left ventricular ejection fraction; LVH, left ventricular hypertrophy.

**Figure 5.**
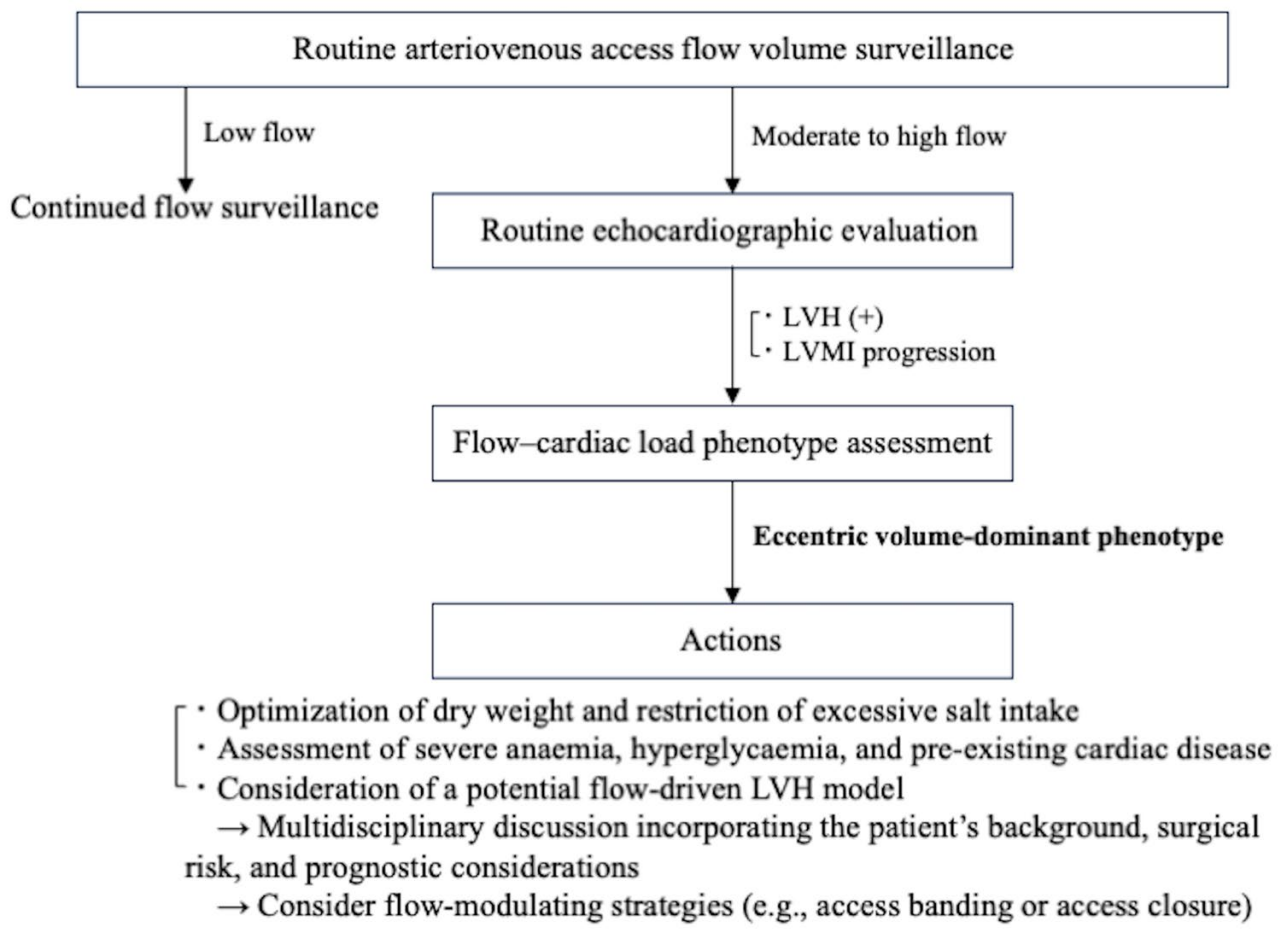
Hypothesis-based, flow-guided surveillance algorithm for arteriovenous access-related cardiac load. Routine access flow surveillance prompts routine echocardiographic evaluation in moderate-to-high flow, focusing on LVH and/or LVMI progression, followed by flow–cardiac load phenotype assessment and proposed actions (volume optimization, evaluation of anemia/hyperglycaemia and preexisting cardiac disease [2], multidisciplinary review, and consideration of flow-modulating strategies). LVH, left ventricular hypertrophy; LVMI, left ventricular mass index.

We propose the term ‘flow-driven LVH (FD-LVH) model’ to conceptualize the progressive cardiac remodeling associated with chronic access flow exposure. This proposed haemodynamic pathway is illustrated in Figure 3. Furthermore, on the basis of our findings, we created a flow–cardiac load phenotype map that integrates AV access FV, LVMI, and basic echocardiographic parameters, including IVST, PWT, and the left ventricular ejection fraction (Figure 4). This phenotype map primarily distinguishes between ‘eccentric volume-dominant’ and ‘concentric load-dominant’ remodeling patterns. The eccentric volume-dominant phenotype represents a framework characterized by moderate to high access FV and flow-driven LV remodeling. This phenotype can be further subdivided into an adaptive phase (‘high-flow compensated’) and a maladaptive or decompensation-prone phase (‘high-flow decompensation risk’). In such patients, the potential relevance of flow-modulating approaches, including access banding or access closure, may be considered in future studies. In contrast, the concentric load-dominant phenotype reflects pressure-dominant remodeling driven by conditions, such as hypertension or aortic stenosis, where the degree of LVH cannot be adequately explained by access flow alone. Similarly, we described a ‘low-flow/high-LVMI’ phenotype. This phenotype corresponds to cases attributable to heterogeneous and cumulative contributors, including obesity, diabetes mellitus, valvular heart disease (e.g., aortic or mitral regurgitation), ventricular septal defect, and preexisting cardiac disease [39]. Notably, as expected, these phenotypes may also be affected by volume overload unrelated to access flow (e.g., overestimation of dry weight, excessive salt intake, and valvular heart disease). Finally, we also propose a flow-guided surveillance algorithm to support clinical decision-making (Figure 5). We anticipate that this conceptual framework may provide clinicians and researchers with a practical classification system and a reference for individualized management strategies in patients on hemodialysis.

LVH and its progression are well-established independent risk factors for cardiovascular events in patients receiving maintenance hemodialysis [19–21]. Conversely, regression of LVH has been associated with improvements in cardiovascular outcomes, including reduced cardiovascular mortality in this population. A growing body of evidence suggests that targeted interventions—particularly those aimed at reducing AV access flow or closing AVF—can lead to partial or complete regression of LVH [40]. Although our cohort was restricted to patients on maintenance hemodialysis, the flow-driven LVH model that we propose may also be relevant to kidney transplant recipients in whom a functioning AVF frequently persists. These findings may inform the controversy surrounding post-transplant AVF ligation and flow modulation as a strategy for LVH regression. These observations underscore the potential benefit of individualized management of AV access flow to mitigate cardiac stress in dialysis patients. Current clinical guidelines reflect this complexity. According to the 2018 Clinical Practice Guidelines issued by the European Society for Vascular Surgery [41], the minimum threshold for adequate access blood flow is 0.3 L/min, with an ideal target of at least 0.5 L/min to ensure sufficient dialysis delivery. However, the definition of ‘high-flow’ access remains ambiguous. The Vascular Access Society defines high-flow AVFs as those with a flow rate between 1.0 and 1.5 L/min and a flow rate-to-cardiac output ratio exceeding 20% [42]. In such cases, routine monitoring with access flow measurements, echocardiography, and clinical assessment for signs of high-output heart failure is recommended. For patients with increasing access flow or clinical indicators of cardiac decompensation, timely interventions—such as banding, flow-reduction surgery, or AVF ligation—should be considered. Importantly, our study demonstrated a linear, positive association between FV and LVMI, even within ranges traditionally considered acceptable for dialysis adequacy. Exploratory findings from the present study suggest that LVMI increases progressively even below traditional ‘high-flow’ cutoffs (1.0–1.5 L/min), warranting investigation of whether lower individualized thresholds may reduce cardiac remodeling. If confirmed in longitudinal or interventional studies, proactive flow adjustment strategies (e.g., banding, staged flow modulation, digital flow-guided surveillance) may emerge as a targetable pathway for LVH regression. On the basis of these findings, individualized access flow targets that balance dialysis adequacy and cardiovascular risk may be warranted, even in asymptomatic patients. In addition, the potential integration of continuous flow monitoring systems or AI-driven digital twins may enable real-time prediction of LV remodeling trajectories.

Future studies should move beyond cross-sectional associations toward prospective, longitudinal, and interventional frameworks that treat AV access flow as a dynamic, modifiable cardio-renal exposure rather than a static adequacy parameter. Recommended research directions include (i) time-resolved phenotyping of cumulative access flow burden using repeated Doppler, echocardiography, and volume status measures to model nonlinear and threshold effects on LV remodeling; (ii) outcome-driven trials testing proactive, flow-guided interventions (e.g., staged banding or precision flow modulation) in asymptomatic patients with progressive LVH despite ‘acceptable’ access flows; (iii) integration of access flow into multimodal risk prediction, combining haemodynamics, fluid metrics, biomarkers, and imaging within machine learning or digital-twin architectures to forecast LVH trajectories and cardiovascular events; and (iv) post-transplant translational studies evaluating whether persistent fistula flow sustains maladaptive remodeling and whether selective ligation reverses the risk. These directions would recalibrate vascular access surveillance from a dialysis-centric paradigm toward a cardio-protective, personalized flow target strategy, with direct implications for guideline development and global clinical practice.

## Limitations

This study has several limitations. First, because this was a multicenter study, LVMI and FV were obtained using echocardiography and Doppler ultrasound that were performed by different clinicians using different machines at each participating center. As a result, inter-observer variability and equipment-related differences may have affected the absolute values of these parameters. FV was calculated as the average of multiple Doppler recordings at each examination, which is likely to have reduced random measurement error, although inter-observer variability cannot be completely eliminated. Second, because the study was limited to three centers in a single geographic region of Japan, generalizability may be restricted. Third, the cross-sectional design precludes causal inference or assessment of longitudinal changes. Finally, residual or unmeasured confounding cannot be fully excluded, despite multivariate adjustment. One potential confounder that was not accounted for in this study is fluid overload, including interdialytic weight gain [43,44], which may have contributed to an overestimation of the association between FV and LVMI.

In conclusion, among patients who had been receiving maintenance hemodialysis for more than 1 year, higher AV access flow was independently associated with increased LVMI, whereas dialysis vintage was not. No statistically significant interaction between FV and dialysis vintage was identified. These observations indicate that AV access flow contributes to cardiac remodeling independent of the dialysis duration and may represent a modifiable driver of cardio-renal haemodynamic burden. Incorporating access flow metrics into future predictive models of LVH risk could improve cardiovascular risk stratification in hemodialysis populations. These results support the consideration of individualized surveillance thresholds and the potential evaluation of flow reduction strategies in prospective outcome-based studies.

## Data Availability

The datasets generated and analyzed during the current study are not publicly available because of ethical restrictions and institutional policies but are available from the corresponding author upon reasonable request.
